# Association of Police Transport With Survival Among Patients With Penetrating Trauma in Philadelphia, Pennsylvania

**DOI:** 10.1001/jamanetworkopen.2020.34868

**Published:** 2021-01-25

**Authors:** Eric Winter, Allyson M. Hynes, Kaitlyn Shultz, Daniel N. Holena, Neil R. Malhotra, Jeremy W. Cannon

**Affiliations:** 1 Division of Traumatology, Surgical Critical Care and Emergency Surgery, Perelman School of Medicine, University of Pennsylvania, Philadelphia; 2 The West Chester Statistical Institute, Department of Mathematics, West Chester University, West Chester, Pennsylvania; 3 McKenna EpiLog Fellowship in Population Health, University of Pennsylvania, Philadelphia; 4 Department of Neurosurgery, Perelman School of Medicine, University of Pennsylvania, Philadelphia; 5 Leonard Davis Institute of Health Economics, University of Pennsylvania, Philadelphia

## Abstract

**Importance:**

Police in Philadelphia, Pennsylvania, routinely transport patients with penetrating trauma to nearby trauma centers. During the past decade, this practice has gained increased acceptance, but outcomes resulting from police transport of these patients have not been recently evaluated.

**Objective:**

To assess mortality among patients with penetrating trauma who are transported to trauma centers by police vs by emergency medical services (EMS).

**Design, Setting, and Participants:**

This cohort study used the Pennsylvania Trauma Outcomes Study registry and included 3313 adult patients with penetrating trauma from January 1, 2014, to December 31, 2018. Outcomes were compared between patients transported by police (n = 1970) and patients transported by EMS (n = 1343) to adult level I and II trauma centers in Philadelphia.

**Exposures:**

Police vs EMS transport.

**Main Outcomes and Measures:**

The primary end point was 24-hour mortality. Secondary end points included death at multiple other time points. After whole-cohort regression analysis, coarsened exact matching was used to control for confounding differences between groups. Matching criteria included patient age, injury mechanism and location, Injury Severity Score (ISS), presenting systolic blood pressure, and Glasgow Coma Scale score. Subgroup analysis was performed among patients with low, moderate, or high ISS.

**Results:**

Of the 3313 patients (median age, 29 years [interquartile range, 23-40 years]) in the study, 3013 (90.9%) were men. During the course of the study, the number of police transports increased significantly (from 328 patients in 2014 to 489 patients in 2018; *P* = .04), while EMS transport remained unchanged (from 246 patients in 2014 to 281 patients in 2018; *P* = .44). On unadjusted analysis, compared with patients transported by EMS, patients transported by police were younger (median age, 27 years [interquartile range, 22-36 years] vs 32 years [interquartile range, 24-46 years]), more often injured by a firearm (1741 of 1970 [88.4%] vs 681 of 1343 [50.7%]), and had a higher median ISS (14 [interquartile range, 9-26] vs 10 [interquartile range, 5-17]). Patients transported by police had higher mortality at 24 hours than those transported by EMS (560 of 1970 [28.4%] vs 246 of 1343 [18.3%]; odds ratio, 1.86; 95% CI, 1.57-2.21; *P* < .001) and at all other time points. After coarsened exact matching (870 patients in each transport cohort), there was no difference in mortality at 24 hours (210 [24.1%] vs 212 [24.4%]; odds ratio, 0.95; 95% CI, 0.59-1.52; *P* = .91) or at any other time point. On subgroup analysis, patients with severe injuries transported by police were less likely to be dead on arrival compared with matched patients transported by EMS (64 of 194 [33.0%] vs 79 of 194 [40.7%]; odds ratio, 0.48; 95% CI, 0.24-0.94; *P* = .03).

**Conclusions and Relevance:**

For patients with penetrating trauma in an urban setting, 24-hour mortality was not different for those transported by police vs EMS to a trauma center. Timely transport to definitive trauma care should be emphasized over medical capability in the prehospital management of patients with penetrating trauma.

## Key Points

**Question:**

Is police-based transport of patients with penetrating trauma associated with lower 24-hour mortality than emergency medical services (EMS)–based transport?

**Findings:**

In this cohort study of 3313 patients with penetrating trauma, individuals with similar injury mechanism and severity had similar 24-hour mortality when transported by police compared with EMS. Patients with the most severe injuries were more likely to be alive on arrival to the hospital when transported by police.

**Meaning:**

The results of this investigation suggest that police transport is safe and effective for patients with penetrating trauma, with equivalent mortality outcomes compared with traditional EMS transport.

## Teaser

This cohort study assesses mortality among patients with penetrating trauma who are transported to trauma centers by police vs emergency medical services in Philadelphia, Pennsylvania.

## Introduction

Penetrating injuries cause more than 42 000 deaths in the United States annually.^1^ Despite recent advances in hospital-based resuscitation practices associated with improved survival, prehospital deaths from penetrating trauma have increased steadily since 2007.^2,3^ The time required to transport patients with penetrating trauma to the hospital is significantly associated with mortality, and rapid transport should be prioritized over field-based advanced life support interventions in critically injured patients with penetrating trauma.^4,5,6^

Although emergency medical services (EMS) personnel typically perform prehospital medical care and provide rapid transport to the hospital, police officers may also be able to provide safe hospital transport for patients with life-threatening penetrating injuries. At a time when social activists are pushing to redefine the scope and objective of policing activities, a first-responder care role for police officers may also build trust between local communities and law enforcement officials.^7^

In 1996, a policy directive in Philadelphia, Pennsylvania, instructed police officers to transport patients with serious penetrating wounds directly to accredited trauma centers and advised that transport should not be delayed to await the arrival of EMS.^7,8,9,10^ The Philadelphia Police Department has since become an essential partner in the care of individuals with penetrating trauma in the city and a model for other police transport programs throughout the country.^10,11,12^ Although other cities (including Chicago, Illinois; Cleveland, Ohio; Detroit, Michigan; and Sacramento, California) have subsequently implemented policies permitting police transport of injured individuals, Philadelphia remains the only major urban center to routinely use this practice.^7,10,12,13^

Before such policies are implemented more broadly, patient volumes and outcomes should be assessed in detail. Past work has suggested that patients with penetrating trauma transported by police have shorter prehospital times relative to those transported by EMS.^8^ Other studies have shown that risk-adjusted mortality is not different between patients transported by police compared with those transported by EMS.^8,9,10^ Based on this evidence, the rate of police transport of patients with penetrating trauma in Philadelphia has significantly increased in recent years. The present study hypothesizes that patients with similar injuries transported to level I and level II trauma centers in Philadelphia by police have lower 24-hour mortality than patients transported by EMS.

## Methods

### Sample Selection and Definitions

**Figure 1.**
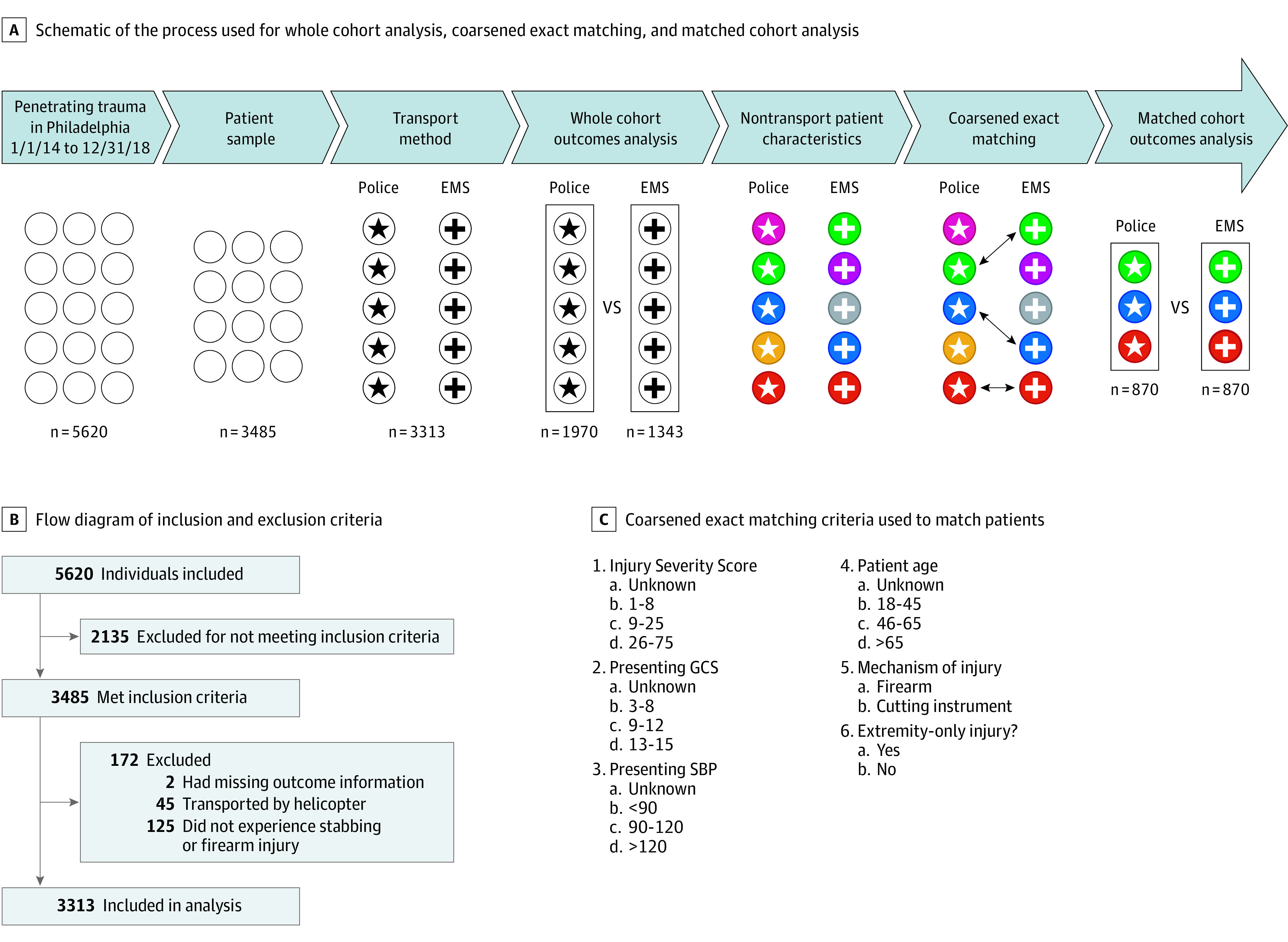
Overview of Patient Matching and Analysis A, Schematic of the process used for whole cohort analysis, coarsened exact matching, and matched cohort analysis. B, Record of patients removed from sample before analysis. C, Coarsened exact matching criteria used to match patients. EMS indicates emergency medical services; GCS, Glasgow Coma Scale; and SBP, systolic blood pressure.

This study followed the Strengthening the Reporting of Observational Studies in Epidemiology (STROBE) reporting guidelines. Patients with penetrating trauma transported by police or EMS directly to a level I or a level II trauma center in Philadelphia from January 1, 2014, to December 31, 2018, were identified in the Pennsylvania Trauma Outcomes Study (PTOS) registry (Figure 1A).^14^ Patients younger than 18 years, pregnant patients, incarcerated patients, those transported by private vehicle or by walk-in, and those transferred from other facilities were excluded (Figure 1B). This study was approved by the University of Pennsylvania institutional review board with a waiver of informed consent because this project involved secondary analysis of existing data, with all human protections and data safety measures in place.

Patients were divided into cohorts based on mode of transport (police vs EMS). Emergency medical services was defined as ground transport by either fire rescue paramedics or other non–fire rescue ambulance. Demographics, injury characteristics, and presenting physiological characteristics were compared between cohorts. Demographics included sex, race/ethnicity, and age. Injury characteristics included Injury Severity Score (ISS; range, 1-75, where 1 indicates a minor injury in a single body region and 75 indicates severe injuries in 3 separate body regions or ≥1 unsurvivable injury), penetrating mechanism, and isolated extremity injury. Penetrating mechanism was defined as an injury with a firearm or cutting instrument, while an isolated extremity injury was defined as patients with only an ISS in body region 5 (extremity) and no other region. Presenting physiological characteristics included Glasgow Coma Scale (GCS) score (range, 3-15, where 3 indicates absent eye opening and no verbal or motor response and 15 indicates spontaneously open eyes with normal verbal and motor function) and initial systolic blood pressure (SBP).

### Matched Cohort Creation

After initial whole cohort analysis, patient characteristics including age, mechanism and location of injury, ISS, presenting GCS score, and SBP were used as matching criteria for coarsened exact matching (Figure 1C). Matching criteria were chosen based on prior work associating these factors with patient outcomes in penetrating trauma.^15,16,17,18^ Data were manually partitioned (“coarsened”) to create discrete categories within each matching criterion.

Matches were sought between patients with different transport methods (police vs EMS) but with an otherwise identical combination of matching criteria strata. Unmatched patients were removed from the data set and were not subjected to further analysis. The resulting matched groups differed only with respect to transport method and had otherwise matched patient composition.

### Sensitivity Analysis and Subgroups

Patients declared dead on arrival were identified for sensitivity analysis to evaluate if removal of these patients from the cohort altered the study findings. Subgroups were defined for patients with different levels of ISS and different types of EMS transport. Injury Severity Scores were divided into low (ISS, 1-8), moderate (ISS, 9-25), and high (ISS, 26-75) levels of injury. Emergency meical services transport was divided into those transported by fire rescue and ambulance.

### Statistical Analysis

Transport volume was assessed using descriptive statistics and linear regression over time. For patient characteristics, categorical variables were compared using the χ^2^ test, and continuous variables were evaluated using the Wilcoxon-Mann-Whitney test.

Whole cohort mortality at 24 hours (primary end point) and all other times was evaluated with univariate logistic regression. Matched cohort mortality at all time points was evaluated by use of the exact McNemar test^19^ and also by use of conditional logistic regression to permit explicit control of the matching terms.^20^ Kaplan-Meier analysis was used to compare outcomes over time in both the whole and matched cohorts.

Data were assessed using SAS, version 9.4 (SAS Institute Inc),^21^ R, version 3.5.2 (R Foundation for Statistical Computing),^22^ and STATA, version 15 (StataCorp LLC). Matching was completed using the MatchIt package,^23^ and the exact McNemar test was completed using the exact2x2 package^24^ in R. Conditional logistic regression was performed with the STATA clogit package. All *P* values were from 2-sided tests and results were deemed statistically significant at *P* < .05.

## Results

From 2014 to 2018, a total of 5620 patients with penetrating trauma were included in the PTOS data set—of these, 3485 met initial inclusion criteria, and 3313 were considered for analysis after detailed data review. A total of 3013 patients (90.9%) were men, and the median age was 29 years (interquartile range [IQR], 23-40 years). Of a total of 1343 (40.5%) in the EMS cohort, 1205 patients (36.4%) were transported directly to a trauma center by fire rescue and 138 patients (4.2%) were transported by ambulance. During this time, 1970 patients (59.5%) were transported by police; the annual rate of police transport was significantly higher than EMS transport (median, 382 of 651 [58.7%; IQR, 57.1%-60.6%] vs 269 of 651 [41.3%; IQR, 39.4%-42.9%]; *P* = .002). During the course of the study, the number of EMS transports remained relatively constant (from 246 patients in 2014 to 281 patients in 2018; *P* = .44), while the number of police transports increased significantly (from 328 patients in 2014 to 489 patients in 2018; *P* = .04) (eFigure 1 in the Supplement).

### Characteristics of Patients

In the prematch sample (n = 3313), the median ISS was 10 (IQR, 6-25), the median GCS score was 15 (IQR, 6-15), and the median SBP was 120 mm Hg (IQR, 82-142 mm Hg). Patients transported by police (n = 1970) had a median age of 27 years (IQR, 22-36 years), a median ISS of 14 (IQR, 9-26), a median GCS score of 15 (IQR, 3-15), and a median SBP of 112 mm Hg (IQR, 70-140 mm Hg); 1741 (88.4%) had been injured by a firearm. Patients transported by EMS (n = 1343) had a median age of 32 years (IQR, 24-46 years), a median ISS of 10 (IQR, 5-17), a median GCS score of 15 (IQR, 14-15), and a median SBP of 126 mm Hg (IQR, 98-145 mm Hg); 681 [50.7%] had been injured by a firearm. These values were all significantly different between the 2 cohorts (all *P* < .001) (Table 1).

**Table 1.** Characteristics of Patients

Characteristic	Whole cohort, No. (%)		*P* value	Matched cohort, No. (%)		*P* value
	Police (n = 1970)	EMS (n = 1343)		Police (n = 870)	EMS (n = 870)	
Sex						
Male	1837 (93.3)	1176 (87.6)	<.001^a^	794 (91.3)	779 (89.5)	.10
Female	133 (6.8)	167 (12.4)		76 (8.7)	91 (10.5)	
Race/ethnicity						
White	97 (4.9)	224 (16.7)	<.001^a^	48 (5.5)	110 (12.6)	<.001^a^
Black	1523 (77.3)	878 (65.4)		666 (76.6)	619 (71.2)	
Hispanic	172 (8.7)	146 (10.9)		74 (8.5)	83 (9.5)	
Asian	13 (0.7)	13 (1.0)		7 (0.8)	7 (0.8)	
Other or unknown	165 (8.4)	82 (6.1)		75 (8.6)	51 (5.9)	
Age, y^b^						
Unknown	69 (3.5)	16 (1.2)	<.001^a^	14 (1.6)	14 (1.6)	.17
18-45	1669 (84.7)	995 (74.1)		734 (84.4)	734 (84.4)	
46-65	216 (11.0)	292 (21.7)		113 (13.0)	113 (13.0)	
>65	16 (0.8)	40 (3.0)		9 (1.0)	9 (1.0)	
Mechanism of injury^b^						
Firearm	1741 (88.4)	681 (50.7)	<.001^a^	663 (76.2)	663 (76.2)	>.99
Cutting instrument	229 (11.6)	662 (49.3)		207 (23.8)	207 (23.8)	
Extremity-only injury^b^						
Yes	133 (6.8)	97 (7.2)	.42	64 (7.4)	64 (7.4)	>.99
No	1837 (93.3)	1246 (92.8)		806 (92.6)	806 (92.6)	
ISS^b^						
Unknown	2 (0.1)	0	<.001^a^	0	0	.39
1-8	375 (19.0)	496 (36.9)		233 (26.8)	233 (26.8)	
9-25	1052 (53.4)	625 (46.5)		443 (50.9)	443 (50.9)	
26-75	541 (27.5)	222 (16.5)		194 (22.3)	194 (22.3)	
Presenting GCS score^b^						
Unknown	71 (3.6)	27 (2.0)	<.001^a^	20 (2.3)	20 (2.3)	.78
3-8	568 (28.8)	265 (19.7)		223 (25.6)	223 (25.6)	
9-12	58 (2.9)	27 (2.0)		13 (1.5)	13 (1.5)	
13-15	1273 (64.6)	1024 (76.3)		614 (70.6)	614 (70.6)	
Presenting SBP, mm Hg^b^						
Unknown	49 (2.5)	25 (1.9)	<.001^a^	15 (1.7)	15 (1.7)	.93
<90	602 (30.6)	270 (20.1)		218 (25.1)	218 (25.1)	
90-120	486 (24.7)	308 (22.9)		194 (22.3)	194 (22.3)	
>120	833 (42.3)	740 (55.1)		443 (50.9)	443 (50.9)	

Abbreviations: EMS, emergency medical services; GCS, Glasgow Coma Scale; ISS, Injury Severity Score; SBP, systolic blood pressure.

^a^ Statistically significant difference between groups.

^b^ Matching criteria.

### Whole Cohort Outcomes

In the prematch unadjusted sample, patients with penetrating trauma transported by police had significantly higher mortality at 24 hours than those transported by EMS (560 of 1970 [28.4%] vs 236 of 1343 [17.6%]; odds ratio [OR], 1.86; 95% CI, 1.57-2.21; *P* < .001) and at all other time points (eFigure 2A and eTable 1 in the Supplement). On Kaplan-Meier analysis, 28-day survival was significantly lower for police-transported patients, with the greatest divergence in survival occurring within the first 24 hours (eFigure 3A in the Supplement). Sensitivity analysis conducted after excluding patients dead on arrival in the police cohort (n = 334) and EMS cohort (n = 150) likewise demonstrated higher mortality in police-transported patients. No patients identified as having an isolated extremity injury expired before or during hospitalization in either transport group (police, 133; and EMS, 97). On subgroup analysis, patients transported by police were also found to have significantly greater mortality than patients transported by fire rescue or ambulance, at all studied time points (eTable 2 and eTable 3 in the Supplement).

Injury severity scores for patients transported by police were significantly higher than those of patients transported by EMS. Patients transported by police with low (ISS, 1-8) and moderate (ISS, 9-25) injury severity had significantly higher 24-hour mortality compared with patients transported by EMS. This difference was not observed for patients with severe injuries (ISS, 26-75) (eFigure 2B and eTable 4 in the Supplement). There was no difference in mortality on arrival between patients transported by police vs EMS at any ISS level. The association between ISS and mortality was also assessed at other time points (eTable 4 in the Supplement).

### Matched Cohort Outcomes

**Figure 2.**
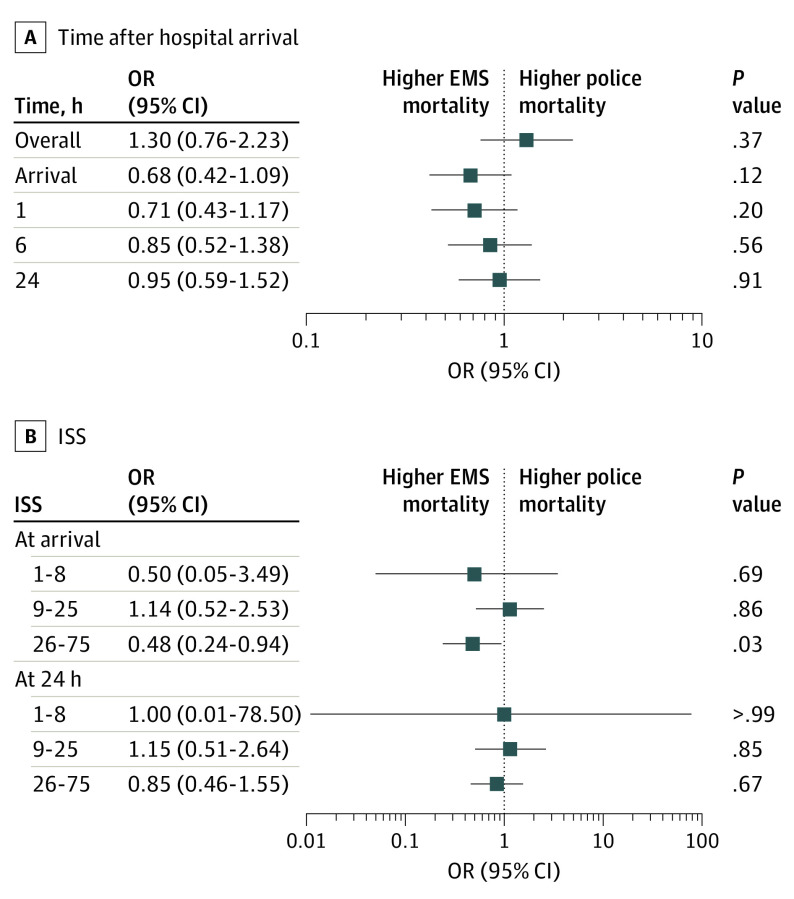
Matched Cohort Outcomes A, Odds ratios (ORs) and 95% CIs for matched cohort patient outcomes at several different time points after trauma center arrival. B, ORs and 95% CIs for matched cohort patient outcomes with different Injury Severity Scores (ISS) at several different time points after trauma center arrival.

Coarsened exact matching identified 870 patients in each transport group (police vs EMS) with an identical combination of matching criteria (Table 1). Patients with penetrating trauma transported by police did not have significantly different mortality from those transported by EMS at 24 hours (210 [24.1%] vs 212 [24.4%]; OR, 0.95; 95% CI, 0.59-1.52; *P* = .91) (Figure 2A; Table 2). This finding was robust to explicit control of the matching terms using conditional logistic regression. Likewise, no significant difference in mortality between the police and EMS cohorts was observed for matched patients overall (238 [27.4%] vs 230 [26.4%]; *P* = .37), on arrival (123 [14.1%] vs 138 [15.9%]; *P* = .12), or at 1 hour (149 [17.1%] vs 161 [18.5%]; *P* = .20) or 6 hours (187 [21.5%] vs 193 [22.2%]; *P* = .56) after arrival. On Kaplan-Meier analysis, 28-day survival was not different between transport groups (eFigure 3B in the Supplement). Sensitivity analysis excluding matched patients who were dead on arrival after police transport (n = 123) or EMS transport (n = 138) likewise showed no difference in mortality (OR, 1.55; 95% CI, 0.88-2.77; *P* = .14). On subgroup analysis dividing patients transported by EMS into those transported by fire rescue and ambulance, there was no significant difference in mortality for either subgroup compared with police transport, at all studied time points (eTable 2 and eTable 3 in the Supplement).

**Table 2.** Matched Cohort Outcomes

Patient mortality	Matched cohort, No. (%)		OR (95% CI)	*P* value
	Police (n = 870)	EMS (n = 870)		
Overall	238 (27.4)	230 (26.4)	1.30 (0.76-2.23)	.37
Arrival	123 (14.1)	138 (15.9)	0.68 (0.42-1.09)	.12
At 1 h	149 (17.1)	161 (18.5)	0.71 (0.43-1.17)	.20
At 6 h	187 (21.5)	193 (22.2)	0.85 (0.52-1.38)	.56
At 24 h	210 (24.1)	212 (24.4)	0.95 (0.59-1.52)	.91

Abbreviations: EMS, emergency medical services; OR, odds ratio.

No difference in 24-hour mortality was observed for matched patients transported by police vs those transported by EMS with any level of injury severity (ISS 1-8: OR, 1.00 [95% CI, 0.01-78.50]; *P* > .99; ISS 9-25: OR, 1.15 [95% CI, 0.51-2.64]; *P* = .85; and ISS 26-75: OR, 0.85 [95% CI, 0.46-1.55]; *P* = .67) (Figure 2B; Table 3). In addition, no difference in mortality on arrival was observed for matched patients with low or moderate injury severity (ISS 1-8: OR, 0.50 [95% CI, 0.05-3.49]; *P* = .69; ISS 9-25: OR, 1.14 [95% CI, 0.52-2.53]; *P* = .86); however, patients with severe injuries had significantly lower mortality on arrival when transported by police (ISS 26-75: 64 of 194 [33.0%] vs 79 of 194 [40.7%]; OR, 0.48 [95% CI, 0.24-0.94]; *P* = .03). The association between ISS and mortality was also assessed at other time points (Table 3).

**Table 3.** Matched Cohort Injury Severity and Mortality

ISS	Matched cohort, No. (%)^a^		Mortality risk for police vs EMS group, OR (95% CI)	*P* value
	Police (n = 870)	EMS (n = 870)		
Overall				
1-8	20 (8.6)	20 (8.6)	1.00 (0.01-78.50)	>.99
9-25	80 (18.1)	76 (17.2)	1.29 (0.60-2.79)	.60
26-75	138 (71.1)	134 (69.1)	1.33 (0.59-3.09)	.57
Arrival				
1-8	16 (6.9)	18 (7.7)	0.50 (0.05-3.49)	.69
9-25	43 (9.7)	41 (9.3)	1.14 (0.52-2.53)	.86
26-75	64 (33.0)	79 (40.7)	0.48 (0.24-0.94)	.03^b^
At 1 h				
1-8	19 (8.2)	20 (8.6)	NA	NA
9-25	48 (10.8)	53 (12.0)	0.72 (0.33-1.56)	.47
26-75	82 (42.3)	88 (45.4)	0.74 (0.37-1.45)	.43
At 6 h				
1-8	20 (8.6)	20 (8.6)	1.00 (0.01-78.50)	>.99
9-25	59 (13.3)	65 (14.7)	0.63 (0.25-1.47)	.33
26-75	108 (55.7)	108 (55.7)	1.00 (0.54-1.87)	>.99
At 24 h				
1-8	20 (8.6)	20 (8.6)	1.00 (0.01-78.50)	>.99
9-25	71 (16.0)	69 (15.6)	1.15 (0.51-2.64)	.85
26-75	119 (61.3)	123 (63.4)	0.85 (0.46-1.55)	.67

Abbreviations: EMS, emergency medical services; ISS, Injury Severity Score; NA, not applicable.

^a^ ISS 1 to 8 (n = 233), ISS 9 to 25 (n = 443), and ISS 26 to 75 (n = 194).

^b^ Statistically significant difference between groups.

## Discussion

This retrospective analysis demonstrates that police officers in Philadelphia transport most patients with penetrating trauma in the city, and between 2014 and 2018, police transport volume increased significantly while EMS transport volume remained relatively unchanged. Patients transported by police were younger, more likely to have been injured by a firearm, more severely injured, and more likely to have hypotension on hospital arrival compared with those transported by EMS. Patients transported by police had significantly higher unadjusted mortality at 24 hours and other secondary time points, relative to those transported by EMS.

Coarsened exact matching was used to attenuate demographic differences between transport groups and control variables associated with injury mechanism and severity. Analysis of matched cohorts revealed no difference in mortality between transport groups at any studied time point. Severely injured patients (ISS 26-75) transported by police were more likely to be alive on arrival to a designated trauma center compared with severely injured patients transported by EMS.

These results are largely consistent with those of prior investigations assessing police vs EMS transport of patients with penetrating trauma.^8,9,10^ One study focused on individuals with proximal penetrating trauma in Philadelphia, and found that overall mortality was significantly higher for patients transported by police compared with those transported by EMS.^9^ Risk-adjusted models showed no difference in mortality between transport groups and subgroup analysis suggested that patients with severe injuries (ISS 15-75) transported by police had lower overall mortality than patients with severe injuries transported by EMS. A nationwide study comparing 2467 police transports and 86 097 EMS transports of patients with penetrating trauma from 2010 to 2012 similarly found no difference in overall survival on risk-adjusted analysis.^10^

Despite evidence demonstrating similar outcomes for patients transported by police and EMS, police transport remains relatively uncommon nationally. Analysis of the National Trauma Data Bank found that only 3% of patients with penetrating trauma were transported by police, and 88% of these transports occurred in Philadelphia (61%), Sacramento (21%), or Detroit (6%).^10^ Although a growing number of cities recognize the utility and safety of this approach, potential barriers to widespread adoption of police transport policies include logistical complexity, officer safety concerns, and scope-of-work issues.^7^

Even though police transport may not be feasible in every area, some locations (such as New York, New York; Chicago, Illinois; and Los Angeles, California) have relatively higher rates of gun violence and longer travel times to designated trauma centers.^7,25,26^ Evidence suggests that “trauma deserts” in these cities are more likely to affect predominately Black neighborhoods, suggesting a possible disparity in access to timely trauma care.^26^ In such areas, law enforcement first responders may be able to improve outcomes by transporting patients with penetrating trauma to trauma centers without waiting for paramedics to arrive and administer potentially ineffective, nonhemostatic interventions at the scene.^6,7^

Police transport policies may also have an impact beyond improving outcomes for patients with penetrating trauma. Evidence from past investigations and the present work suggests that most patients transported by police are young Black men, who are often injured in areas with historically adversarial relationships between residents and law enforcement.^7,27,28^ In some cases, injured Black individuals transported by police report appreciating the improved scene safety and expedited hospital transport facilitated by responding officers.^29^ Moreover, police transport may help diffuse scene anxiety and allay panic by responding to bystander requests for immediate assistance.^7,30^ Police transport programs thus have the potential to change established negative perceptions of law enforcement by demonstrating altruistic attributes of the police in their neighborhoods.^7,29^ Although police transport policies should be considered primarily through the lens of patient outcomes, the positive association of such policies with community dynamics may serve to lower actual or perceived barriers to implementation.

### Coarsened Exact Matching

This study used coarsened exact matching. This statistical method retains the full dimensionality of each patient’s information such that patients are paired only when they “exactly match” on all individual matching criteria. Compared with other matching strategies, coarsened exact matching is able to independently control for many potential confounding variables associated with injury mechanism and severity.^31^ The matching criteria used in the present work were selected based on past work correlating patient age, ISS, GCS score, and SBP with patient outcomes.^15,16,17,18^

### Limitations

In addition to the limitations inherent in a retrospective registry-based study, the results of the present work should be interpreted in the context of several other limitations. First, although the PTOS trauma registry represents a rich repository of data fields, geographical injury locations and prehospital transport times are not reliably recorded. This is particularly significant as the concept of police transport hinges on rapid identification and evacuation of patients with penetrating trauma to designated trauma centers. Furthermore, the PTOS registry does not include prehospital interventions that could either improve survival (eg, tourniquet application) or that conversely might delay transport without enhancing survival (eg, intubation). Future prospective work in this area should note both transport times and interventions.^32^

Although the PTOS database does include ISS for each injured body region, it does not contain specific information on the anatomical injuries. This precludes detailed analysis of the association between specific bodily injuries and patient survival. Furthermore, ISS may be inaccurate for patients who die prior to complete imaging or surgical intervention, and because ISS is calculated based on the highest recorded injury severity in 3 different body regions, the ISS for a patient who sustains multiple wounds to a single body region may not reflect the full extent of their injuries. Nonetheless, the incidence of these potential sources of bias should affect both cohorts equally in the present study.

Although this study sought to match comparable patients, it is possible that using additional characteristics such as presence of a prehospital heart rate, preexisting medical conditions, or injury scene distance from a trauma center would result in more perfect matching between transport groups with improved control of confounding variables. Finally, this analysis focused exclusively on patients with penetrating injuries in an urban environment replete with police, EMS crews, and trauma centers. Thus, these findings may not be generalizable to other types of injuries or to other geographical locations such as suburban or rural areas.^7^

## Conclusions

Police transport of patients with penetrating trauma in Philadelphia increased significantly from 2014 to 2018. Patients transported by police were more severely injured and more often hypotensive than patients transported by EMS, yet after controlling for significant differences between groups, patients transported by police had similar mortality. Thus, widespread application of the police transport policy in Philadelphia appears to provide a safe and effective transport modality that can be considered complementary to EMS transport. Patients in other urban locales may benefit from the implementation and broader application of similar policies that encourage police to rapidly transport patients to designated trauma centers without waiting for EMS crews to arrive. Future studies should assess for differences in transport time and the association of prehospital interventions with time to definitive hemostasis and patient survival.

## Notes

Supplement. **eTable 1.** Mortality in the Whole Cohort **eTable 2.** Mortality Comparing Police Transport With the Fire Rescue Subgroup of Emergency Medical Services Transport **eTable 3.** Mortality Comparing Police Transport With the Ambulance Subgroup of Emergency Medical Services Transport **eTable 4.** Mortality in the Whole Cohort Grouped by Injury Severity **eFigure 1.** Police vs Emergency Medical Services (EMS) Transport Over Time **eFigure 2.** Whole Cohort Outcomes **eFigure 3.** Patient Survival for Police vs Emergency Medical Services (EMS) Transport
