# Supplementary material for: Association of Police Transport With Survival Among Patients With Penetrating Trauma in Philadelphia, Pennsylvania
Source: JAMA Netw Open. 2021 Jan 25;4(1):e2034868. doi: 10.1001/jamanetworkopen.2020.34868 (PMC7835719; doi:10.1001/jamanetworkopen.2020.34868)
Supplement: Supplement. — eTable 1. Mortality in the Whole Cohort eTable 2. Mortality Comparing Police Transport With the Fire Rescue Subgroup of Emergency Medical Services Transport eTable 3. Mortality Comparing Police Transport With the Ambulance Subgroup of Emergency Medical Services Transport eTable 4. Mortality in the Whole Cohort Grouped by Injury Severity eFigure 1. Police vs Emergency Medical Services (EMS) Transport Over Time eFigure 2. Whole Cohort Outcomes eFigure 3. Patient Survival for Police vs Emergency Medical Services (EMS) Transport [file jamanetwopen-e2034868-s001.pdf]

Supplementary Online Content

Winter E, Hynes AM, Shultz K, Holena DN, Malhotra NR, Cannon JW. Association of police transport with survival among patients with penetrating trauma in Philadelphia, Pennsylvania. *JAMA Netw Open.* 2021;4(1):e2034868. doi:10.1001/jamanetworkopen.2020.34868

eTable 1. Mortality in the Whole Cohort

eTable 2. Mortality Comparing Police Transport With the Fire Rescue Subgroup of Emergency Medical Services Transport

eTable 3. Mortality Comparing Police Transport With the Ambulance Subgroup of Emergency Medical Services Transport

eTable 4. Mortality in the Whole Cohort Grouped by Injury Severity

eFigure 1. Police vs Emergency Medical Services (EMS) Transport Over Time

eFigure 2. Whole Cohort Outcomes

eFigure 3. Patient Survival for Police vs Emergency Medical Services (EMS) Transport

This supplementary material has been provided by the authors to give readers additional information about their work.

© 2021 Winter E et al. *JAMA Network Open.*

eTable 1. Mortality in the Whole Cohort

		Whole Cohort			
		Police n = 1970	EMS n = 1343	Odds Ratio (95% CI)	P Value
**Patient Mortality**	Overall	620 (31.47)	258 (19.21)	1.93 (1.64-2.28)	**< .001**
	Arrival	334 (16.95)	150 (11.17)	1.62 (1.32-2.00)	**< .001**
	1-Hour	398 (20.20)	175 (13.03)	1.69 (1.39-2.05)	**< .001**
	6-Hour	505 (25.63)	213 (15.86)	1.83 (1.53-2.18)	**< .001**
	24-Hour	560 (28.43)	236 (17.54)	1.86 (1.57-2.21)	**< .001**

Data shown as n (%) and odds ratio (OR) with 95% CI. Bold text denotes statistically significant result.

© 2021 Winter E et al. *JAMA Network Open.*

eTable 2. Mortality Comparing Police Transport With the Fire Rescue Subgroup of Emergency Medical Services Transport

		**Whole Cohort**				**Matched Cohort**			
		Police n = 1970	Fire Rescue n = 1205	Odds Ratio (95% CI)	P Value	Police n = 797	Fire Rescue n = 797	Odds Ratio (95% CI)	P Value
**Patient Mortality**	Overall	620 (31.47)	228 (18.92)	1.97 (1.66-2.34)	**< .001**	198 (24.84)	203 (25.47)	0.82 (0.44-1.49)	.568
	Arrival	334 (16.95)	137 (11.37)	1.59 (1.29-1.97)	**< .001**	110 (13.80)	124 (15.56)	0.65 (0.38-1.09)	.109
	1-Hour	398 (20.20)	159 (13.20)	1.67 (1.36-2.03)	**< .001**	130 (16.31)	144 (18.07)	0.59 (0.32-1.05)	.076
	6-Hour	505 (25.63)	190 (15.77)	1.84 (1.53-2.22)	**< .001**	162 (20.33)	171 (21.46)	0.71 (0.39-1.27)	.272
	24-Hour	560 (28.43)	209 (17.34)	1.89 (1.58-2.26)	**< .001**	175 (21.96)	186 (23.34)	0.69 (0.39-1.19)	.193

Data shown as n (%) and odds ratio (OR) with 95% CI. Bold text denotes statistically significant result.

© 2021 Winter E et al. *JAMA Network Open.*

eTable 3. Mortality Comparing Police Transport With the Ambulance Subgroup of Emergency Medical Services Transport

		Whole Cohort				Matched Cohort			
		Police n = 1970	Ambulance n = 138	Odds Ratio (95% CI)	P Value	Police n = 129	Ambulance n = 129	Odds Ratio (95% CI)	P Value
**Patient Mortality**	Overall	620 (31.47)	30 (21.74)	1.65 (1.09-2.51)	**.014**	34 (26.36)	29 (22.48)	2.67 (0.64-15.61)	.227
	Arrival	334 (16.95)	13 (9.42)	1.96 (1.10-3.52)	**.014**	12 (9.30)	13 (10.08)	0.83 (0.20-3.28)	1.000
	1-Hour	398 (20.20)	16 (11.59)	1.93 (1.13-3.29)	**.009**	17 (13.18)	16 (12.40)	1.25 (0.27-6.30)	1.000
	6-Hour	505 (25.63)	23 (16.67)	1.72 (1.09-2.73)	**.014**	22 (17.05)	22 (17.05)	1.00 (0.30-3.34)	1.000
	24-Hour	560 (28.43)	27 (19.57)	1.63 (1.06-2.51)	**.020**	29 (22.48)	26 (20.16)	1.50 (0.48-5.12)	.607

Data shown as n (%) and odds ratio (OR) with 95% CI. Bold text denotes statistically significant result.

© 2021 Winter E et al. *JAMA Network Open.*

eTable 4. Mortality in the Whole Cohort Grouped by Injury Severity

			Whole Cohort			
			Police n=1970*	EMS n=1343†	Odds Ratio (95% CI)	P Value
**Overall**	**ISS**	1-8	34 (9.07)	22 (4.44)	2.15 (1.23-3.74)	**.006**
		9-25	207 (19.68)	92 (14.72)	1.42 (1.09-1.86)	**.010**
		26-75	379 (70.06)	144 (64.86)	1.27 (0.91-1.77)	.163
**Arrival**	**ISS**	1-8	26 (6.93)	20 (4.03)	1.77 (0.97-3.23)	.060
		9-25	106 (10.08)	47 (7.52)	1.38 (0.96-1.97)	.075
		26-75	202 (37.34)	82 (37.39)	1.00 (0.72-1.38)	.989
**1-Hour**	**ISS**	1-8	31 (8.27)	22 (4.44)	1.94 (1.10-3.41)	**.020**
		9-25	126 (11.98)	61 (9.76)	1.26 (0.91-1.74)	.160
		26-75	241 (44.55)	92 (41.44)	1.14 (0.83-1.56)	.432
**6-Hour**	**ISS**	1-8	33 (8.80)	22 (4.44)	2.08 (1.19-3.63)	**.009**
		9-25	164 (15.59)	77 (12.32)	1.31 (0.98-1.76)	.063
		26-75	308 (56.93)	114 (51.35)	1.25 (0.92-1.71)	.160
**24-Hour**	**ISS**	1-8	34 (9.07)	22 (4.44)	2.15 (1.23-3.74)	**.006**
		9-25	186 (17.68)	83 (13.28)	1.40 (1.06-1.86)	**.016**
		26-75	340 (62.85)	131 (59.01)	1.18 (0.85-1.62)	.323

Data shown as n (%) and odds ratio (OR) with 95% CI. Bold text denotes statistically significant result.
Emergency Medical Services, EMS; Injury Severity Score, ISS
*ISS 1-8 n=375, 9-25 n=1052, 26-75 n=543
†ISS 1-8 n= 496, 9-25 n=625, 26-75 n=222

© 2021 Winter E et al. *JAMA Network Open.*

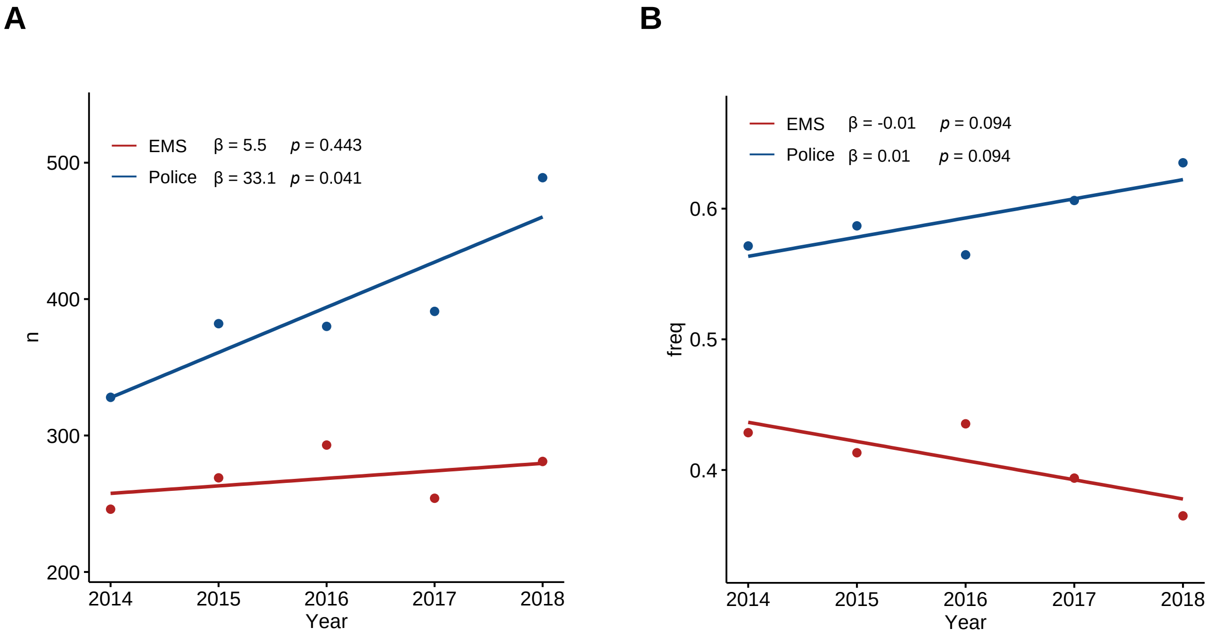

eFigure 1. Police vs Emergency Medical Services (EMS) Transport Over Time

(A) Number of transports in each cohort from 2014 to 2018. (B) Relative frequency of police vs EMS transport for each year from 2014 to 2018.

© 2021 Winter E et al. *JAMA Network Open.*

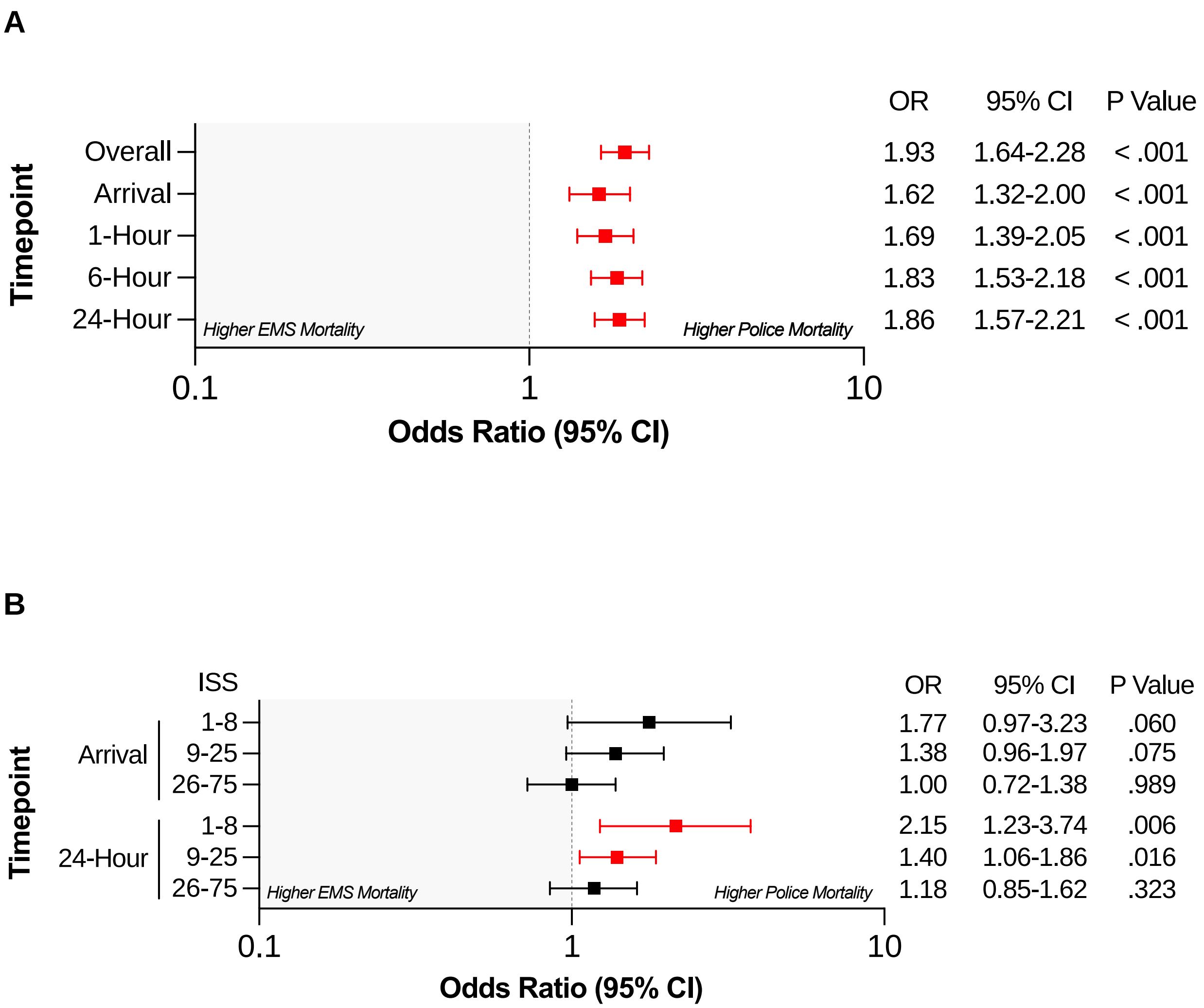

eFigure 2. Whole Cohort Outcomes

(A) Odds ratios and 95% confidence intervals for whole cohort patient outcomes at several different time points after trauma center arrival. (B) Odds ratios and 95% confidence intervals for whole cohort patient outcomes with different injury severity scores, at several different time points after trauma center arrival.

© 2021 Winter E et al. *JAMA Network Open.*

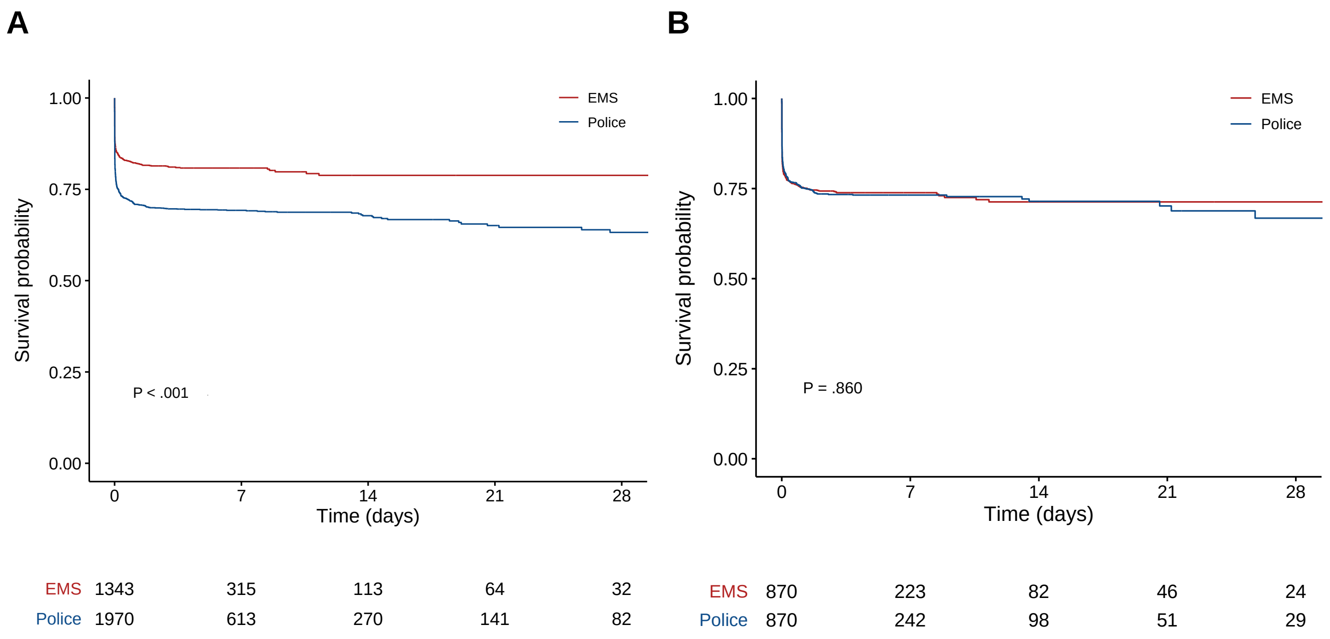

eFigure 3. Patient Survival for Police vs Emergency Medical Services (EMS) Transport

(A) Kaplan-Meier curve comparing unadjusted 28-day survival for the whole cohort. (B) Kaplan-Meier curve comparing unadjusted 28-day survival for the matched cohort.

© 2021 Winter E et al. *JAMA Network Open.*
